# Pediatric Acute Myeloid Leukemia—Past, Present, and Future

**DOI:** 10.3390/jcm11030504

**Published:** 2022-01-19

**Authors:** Dirk Reinhardt, Evangelia Antoniou, Katharina Waack

**Affiliations:** Pediatrics III, Department of Pediatric Hematology, Oncology and Stem Cell Therapy, University Children’s Hospital, University Duisburg, 45147 Essen, Germany; Evangelia.Antoniou@uk-essen.de (E.A.); katharina.waack@uk-essen.de (K.W.)

**Keywords:** acute myeloid leukemia, children, treatment, prognosis

## Abstract

This review reports about the main steps of development in pediatric acute myeloid leukemia (AML) concerning diagnostics, treatment, risk groups, and outcomes. Finally, a short overview of present and future approaches is given.

## 1. Introduction

The treatment of pediatric acute myeloid leukemia (AML) is a success story in improving prognosis. Whereas, in the 1980s, almost all children suffering from AML died, today, up to 75% of the children survive. However, this is only feasible in a well-structured setting of comprehensive diagnostics, intensive therapy, and effective supportive care. This has been achieved by the cooperative study groups in Europe, North America, and Japan. By contrast, even within Europe, the prognosis of children with AML shows an unacceptable level of inequality of survival rates, ranging from less than 50% to 80% [1]. 

The incidence of pediatric AML is about seven per million, with only minor differences between continents or countries. The malignant blasts originate from early hematopoietic progenitors as an evolution from (pre-)leukemia stem cells. External/environmental factors could explain only a tiny percentage. In addition, predisposing syndromes or germline mutations are associated with less than 10% of pediatric AML. During childhood and adolescence, infants less than two years old and adolescents have the highest incidence. Whereas MLL-rearranged leukemia dominates during infancy, the frequency of core-binding leukemia (CBL) and AML associated with mutations, such as NPM 1 or FLT3-ITD, increases by age [2].

**Figure 1:**
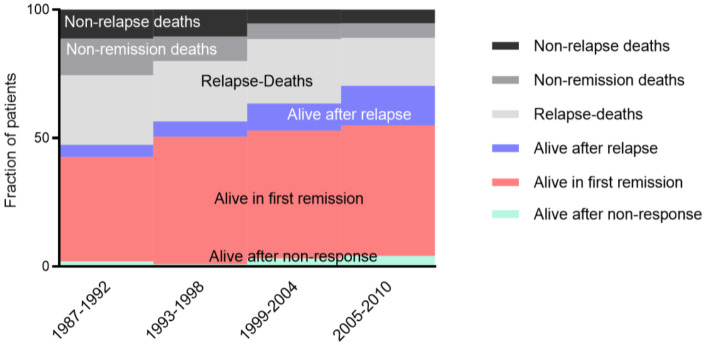
Improvement of outcome in pediatric AML. Continuous increase of survival in first remission (red), following initial non-response (green), and after relapse (blue). In parallel, the treatment-related mortality (black, non-relapse deaths) and non-remission deaths (grey) decreased significantly. This supported the hypothesis that the improved overall survival is based on better treatment and improved supportive care.

Except for acute promyeloblastic leukemia (APL), improved survival has been achieved by using long-known conventional drugs, mainly cytarabine and anthracyclines. Scheduling risk group stratification, modifications of allogeneic stem cell transplant (alloHSCT), and management of complications allowed for curing most children (Figure 1) [3,4]. 

The only new approach within the most recent 20 years was the targeted CD33 antibody gemtuzumab ozogamicin (GO), which showed some advantages in at least one randomized trial, but GO is only approved in North America [5]. 

The successful therapy of APL with all-trans retinoic acid and arsenic-trioxide is a rare example of curing leukemia targeting specifically leukemia-inducing molecular mechanisms and eradicating the leukemic stem cell [5,6]. 

Liposomal drug formulation of daunoribiicn allowed treatment intensification without increasing toxicities, but this has disappeared due to economic reasons, such as a limited pediatric market. The actual approach with a liposomal nanoscale co-formulation of cytarabine and daunorubicin seems to be promising but needs confirmatory trials in pediatric AML and marketing approval thereof. 

Although stem cell transplantation is still an unspecific treatment option with severe acute and long-term side effects, combined with a better risk group stratification, alloHSCT significantly improved survival in children with high-risk (HR) AML [7,8]. 

There are a broad number of new compounds explicitly targeting signaling pathways. However, it is not confirmed in children to what extent these approaches will contribute to curing or be able to reduce toxicities by allowing reduction of treatment intensity of conventional drugs. 

A fast-growing field are immune and cellular therapies, which show promising results in preclinical and early phase clinical trials (mainly in adults).

## 2. Past

Although pediatric AML has been described since the 1900s, a formal classification was established, such as in adults, in 1976 by the French-American-British (FAB)-Classification [9]. There are already six subtypes of AML that have been established and described. The regular introduction of immunophenotyping modified this morphology and cytochemistry-based classification during the 1990s [10,11,12,13]. In the WHO classification in 2001, a shift from morphology to a primarily genetically-based classification has been released and continuously extended. 

Until 1968, the remission rate was inferior, and the median survival was about 1.5 months [14]. With the implementation of an intensified block therapy, including cyclophosphamide and cytarabine, a survival of 9.5 months was achieved, in 1976 [15].

Since 1975 the first clinical trials for pediatric patients with AML were initiated [16,17,18]. Cooperative Study groups have been established: AIEOP (Associazione Italiana di Ematologia e Oncologia Pediatrica), AML-BFM (Berlin, Frankfurt, Münster), NOPHO (Nordic Society for Pediatric Hematology and Oncology), MRC (Medical Research Council), and EORTC (European Organization of Research and Treatment of Cancer), CCG (Childhood Cancer Group), POG (Pediatric Oncology Group; merged in 2000 to COG (Children’s Oncology Group); and SJCRH (St. Jude Children’s Research Hospital). Whereas the MRC conducted combined pediatric and adult trials [19], the AML-BFM 78 study (1978–1983) was a pure pediatric trial to examine the application of two to six courses of daunorubicin, cytarabine, and 6-thioguanine (DAT) after a first induction of the same course [17]. In the AML 10 trial of the MRC group, the comparison of etoposide with thioguanine, as randomization from ADE versus DAT, showed no significant difference [20]. Significant progress in pediatric AML was made by the AML-BFM 83 trial, based on the introduction of block-scheduling. Within this trial, the favorable risk groups of AML with t(8; 21) and, inv(16) have been identified and confirmed in children. The same favorable cytogenetic criteria were confirmed in the MRC AML 10. As adverse characteristics, -5, -7, del(5q), abn(3q), and complex karyotype were documented [20,21].

Although most patients were defined just by morphology, due to the high correlation between FAB M2 with Auer rods and t(8;21), the presence of atypical eosinophils in FAB M4 Eo with inv(16) or, later, FAB M2 and NPM1-mutated AML allowed event-free survival (EFS) rates of 70% and more [22,23]. Interestingly, despite further intensification, this has not changed until today. In the LAME90/91 study, patients were classified into two groups dependent on whole blood count (WBC) and cytogenetics [24]. The standard risk group included t(8;21), inv(16), t(15;17) (defined as FAB M2, M4, and M3), and also patients with <100 000/μL leukocytes, initially. The AML BFM Study Group described hyperleukocytosis as a poor prognostic marker but was not used for risk stratification [25]. 

Within the 20th century, the relevance of anthracycline analogs has been discussed. Randomized trials, such as AML-BFM 98 and MRC 12/15, tested idarubicin versus daunorubicin or mitoxantrone. Significant achievements were the identification of idarubicin as the most effective anthracycline if applied in a 1 to 5 conversion rate compared to daunorubicin [26,27]. 

In the NOPHO-93, all the patients initially underwent the same treatment (ATEDox), but, dependent on the response after the first induction, an extra AM (cytarabine, Mitoxantrone) induction was recommended [28]. Those patients with excess blasts after the AM course received an HA2E course. 

The tested antileukemic drug in the NOPHO 2004 trial was GO and did not show any significant effect on the recurrence rate of leukemia or overall survival (OS) [29]. An extra criterium of this study was the presence of mixed lineage leukemia (MLL) rearrangements other than t(9;11), suppoeritng for the first time the independent involvement of cytogenetics in the risk stratification. During this study, especially in 2009, the criteria for the high-risk (HR) patients were restricted to the poor response [7]. 

The St. Jude AML02 Study stratified the patients into two subgroups according to morphologic and genetic characteristics. Patients were randomized to receive daunorubicin (50 mg/m^2^ on days 2, 4, and 6) and etoposide (100 mg/m^2^ on days 2–6), and high-dose cytarabine (3 g/m^2^ every 12 hours) [30]. 

The Japanese AML99 study (JPLSG) implemented a risk stratification of three groups. The initial stratification was made for the low-risk patients, and including the HR group criteria of WBC (>100.000 µL) and the age of the patients (<2 years). Additionally, the response after the first induction and the karyotype led to the allocation of the patients in the final stratification group. An alloHSCT was indicated only for the intermediate and HR groups, especially for the last group; a “not familiar” donor was suggested [31].

In the AML05 study (JPLSG), the reduced cumulative anthracycline dose (<300 mg/m^2^) was tested in the low and intermediate-risk patients. At the same time, 50% of the etoposide dose was used in the AML99 protocol. A higher incidence of relapse was noticed, but the OS was not influenced [32]. 

In the early 2000s, international cooperative projects, analyzing a larger cohort of patients, finally defined further prognostic factors [4]. This also led to a harmonization of risk group definition worldwide. All major study groups agreed on AML with t(8;21) and inv(16) as a favorable prognostic group that could be cured with chemotherapy only [23,31]. There are still controversies about the definition and post-remission therapy of the patients belonging to the intermediate-risk group. Some groups recommend alloHSCT, while others stick to chemotherapy. In addition, the HR group was not finally defined, hence remaining heterogenous. In addition, in that period, large treatment groups failed to demonstrate the advantage of alloHSCT in the HR group [33,34,35]. 

### 2.1. Hematopoietic Stem Cell Transplantation

AlloHSCT was introduced in the 1980s as post-consolidation therapy in pediatric AML. Although effective in some cases, it never achieved the status as a general standard in contrast to adults. This is explained by the relevant side and long-term effects and the effective chemotherapy in children, which already allowed in the 1990s a long-term survival of about 60% [36,37]. Finally, the improved risk group stratification allowed the identification of those children who benefit. Nevertheless, it is an ongoing process to identify the pediatric AML subgroups who finally benefit from alloHSCT. This includes all associated issues, such as donor selection, prevention and treatment of graft versus host disease, management of virus reactivation, and immune reconstitution.

Between the mid-1980s and the 1990s, progress in pediatric AML was limited. Autologous HSCT or alloHSCT from a matched sibling donor have been introduced to the therapy [38,39]. Although there was a reduction of relapses, this could not be translated to improved OS. Transplant-related mortality counterbalanced the potentially increased antileukemic effect [40]. Considering the significantly higher risk of post-transplant late sequelae, long-lasting controversies about the relevance and importance of alloHSCT in first complete remission occurred [33,40,41,42,43,44]. 

In the AML-BFM 87 study and the MRC AML 12 trial, the response on day 15 after the first induction was added as a criterium for stratification [27,45]. Whereas alloHSCT was not generally recommended in the AML-BFM trial, the British study group limited alloHSCT to the intermediate and poor-risk group [27]. 

### 2.2. CNS Prophylaxis and Treatment

A monotherapy with intrathecal cytarabine was used as prophylactic therapy in the CCG-2891 and as a treatment twice a week in a total of six doses for the central nervous system (CNS) positive patients [46]. In Italy, the VAPA protocol, the first conducted study in children, also included monotherapy with cytarabine with 12 doses for all included patients [47]. Triple intrathecal therapy with methotrexate, cytarabine, and hydrocortisone was administered in the MCR AML10 and NOPHO 2004 twice a week by CNS positive patients until cerebrospinal fluid (CSF) clearance. The difference was the clearance duration after the extra intrathecal inductions, one and two weeks, respectively, for the MRC and NOPHO treatment groups [29]. 

In the AML-BFM-87 study, prophylactic irradiation was randomized. However, an increased rate of relapses in children without cranial irradiation led to a premature stop [45]. Since then, the AML-BFM studies have included cranial irradiation as a mandatory treatment for all patients, except those who received alloHSCT [48]. In contrast, the CCG and MRC group applied cranial irradiation only if the CSF was not cleared after the intrathecal therapy [35,49]. The standard therapy in the AML02 study of the JPLSG included triple intrathecal therapy in each course but no prophylactic cranial irradiation [31]. Based on the more intensive and CNS-effective chemotherapy, since 2012 the AML-BFM group no longer applied prophylactic cranial irradiation. Only in patients with CNS involvement is irradiation still recommended [50].

To summarize, different intrathecal therapies, such as monotherapy or triple treatment, were administered from the studies mentioned above. Cranial irradiation was excluded from the standard treatment and is now only recommended for children with CNS involvement. 

### 2.3. Development of Minimal Residual Disease (MRD) Diagnostics

In parallel to the intensified therapy, the relevance of genetic risk groups and treatment response became obvious. Improved techniques, such as multicolor immunophenotyping and quantitative PCR, allowed a more precise response detection. 

Detection of MRD by multicolor flow cytometric immunophenotyping started during the 1990s. The leukemic blasts were selected via different antigens (CD45 and CD34, CD117, CD13, CD15, CD33, etc.). 

Langebrake et al. defined different response measurements within AML subtypes, including the prognostics relevance [51]. In contrast, in the SJCRH AML02 study, a higher incidence of relapse was noticed in patients with MRD 1% after the first induction and >0.1% after the second induction. MRD was characterized as a poor prognostic factor EFS and OS [52]. 

In the NOPHO 2004 study, a difference in the EFS, but not in OS, comparing the MRD positive with the morphologic positive patients was noticed [53]. 

Within the Dutch Childhood Oncology Group (DCOG) ANLL, 97, and the MRC 12 trials, MRD levels measured by flow were prognostically favorable for the patients achieving MRD negativity after the first/second induction [54].

Polymerase chain reaction (PCR) is also used to detect MRD for different fusion transcripts. The correlation of expression with the clinical progress of the patients is supported in many studies. The data presented are promising for using this method as a sensitive diagnostic tool. The studies include a small number of patients for the specific subpopulations in the pediatric population, such as t(8;21), in(16) [55].

## 3. Present

The cooperative trial groups achieved significant improvements in overall survival. Table A1 (Appendix A) summarizes recent results, showing similar despite different chemotherapy schemes. 

The analysis of the AML-BFM trials between 1993 and 2010 revealed a continuous improvement of OS but limited progress of EFS [50]. This suggests that 2nd line treatment plays a relevant role in explaining the increasing gap between EFS and OS [56]. 

Treatment regimens include initial double induction and 2- or 3-consolidation blocks. Even if the definitions vary, it is evident that about 60% of children with AML can be cured by chemotherapy only. On the other hand, a significant achievement within the ongoing trials was the establishment of a risk group dependent indication for alloHSCT in the 1st CR [4]. 

Along with the improvements in the transplant procedure and the option to rescue refractory AML, EFS improved [8]. A condition is supportive care to prevent and manage expected complications and more precise diagnostics to allow a genetic and response-based stratification [4]. 

### 3.1. alloHSCT

The AML-BFM Study group and the NOPHO proved that alloHSCT in the 1st CR of high-risk pediatric AML improves EFS, which is not significantly lower than intermediate-risk (IR) or standard risk (SR) [7,8]. However, due to the intensified therapy, including alloHSCT, the salvage treatment is ineffective, compared to IR/SR, resulting in a still inferior OS. 

In case of relapse, all patients indicate alloHSCT in the 2nd CR. Although the prognostic characteristics of relapsed AML are impaired, the survival rate has been maintained or improved [56,57]. 

This allows treating the “right” patient group with the “right” intensity. In particular, the precise definition of the high-risk group by genetics and response associated with the indication of alloHSCT in the 1st CR eliminated significant differences in EFS. 

To achieve this, the results of the AML-BFM 2004 trial have been re-analyzed. Based on the genetic characteristics and augmented by the treatment response to the 1st and 2nd induction, three risk groups could be defined [58]. 

In the AML-BFM 2012 Registry, this risk group stratification has been implemented. The NOPHO Group and others have published similar reports [7]. 

**Figure 2:**
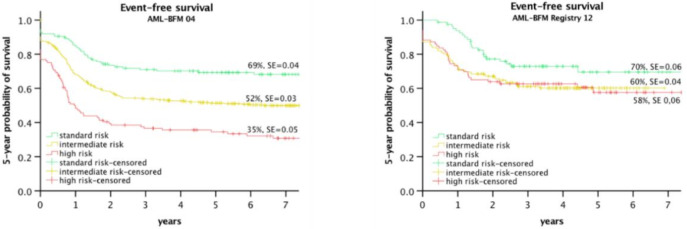
EFS of risk groups in Study AML-BFM 2004 [58] and AML-BFM registry 2012 [8].

Figure 2 shows the improvement of the HR Group in the AML-BFM 2012 Registry, including the suspension of significant differences between the risk groups.

**Figure 3:**
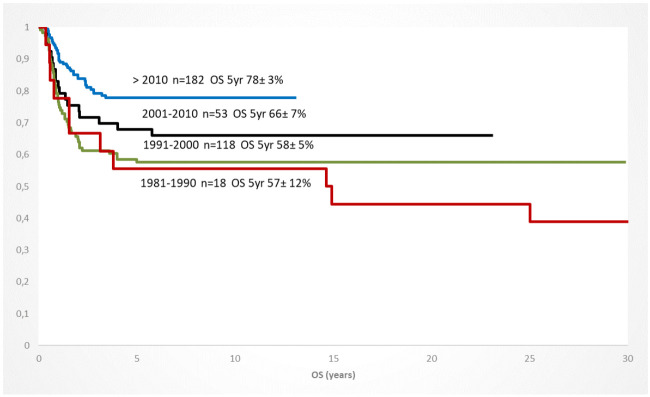
Increase of overall survival of children with AML and HSCT in CR1 (any donor/conditioning) since 1981; 10 year-periods (AML-BFM trials).

In addition to the improved risk group stratification, the selection of conditioning regiments, preparation of the transplanted stem cells, the donor identification and availability, and the graft-versus-host prophylaxis significantly contributed to a better outcome in the 1st/2nd CR but also in children with a refractory AML [59]. Within the de-novo AML patients who were transplanted in the 1st CR, the OS increased continuously between 1981 and 2019, documenting the improvement of the treatment approach over time (Figure 3).

Most groups have accepted the standard for myeloablative conditioning with busulfan, melphalan, and cyclophosphamide. Earlier studies with less intensive conditioning (busulfan/cyclophosphamide) resulted in unacceptably high relapses [37]. However, concerns about severe acute toxicities, especially in adolescents, supported the application of alternative regimens, such as treosulfan, fludarabine, and thiotepa [60,61]. Other regiments include clofarabine, busulfan, and fludarabine [59]. 

Regarding stem cell selection, the CD3/CD19-depleted graft transplantation of bone marrow or apheresis cells is the most widely used approach. The donor selection included matched sibling donors (MSD) or matched unrelated donors (MUD), defined as a 9/10 or 10/10 allele match for the HLA loci A, B, C, DR, and DQ, as determined by molecular 4-digit high-resolution typing. For the HR patients without a matched donor, a haploidentical donor is accepted [59]. 

An unexpected, good outcome has been achieved in children with refractory AML, who got fludarabine/amsacrine (FLAMSA) and reduced conditioning (TBI/DLI). The reported 4-years EFS of 41% seems to be promising because, in the past, almost all patients of this cohort died [59].

### 3.2. Diagnostics

The diagnostic of pediatric AML requires morphology, immunophenotyping, and comprehensive cyto- and molecular genetics of the leukemic blasts. All available methods, such as multicolor flow with at least eight colors, panel-next-generation sequencing (NGS), and RNA seq, must be integrated. In general, the risk groups definition can be based mainly on genetics augmented by response measurement by flow and morphology (AIEOP/AML-BFM/FRANCE/UK/COG/Japan), or visa-versa, preferentially MRD-driven augmented by genetics (NOPHO) [62].

Table 1 shows the definition of risk groups according to genetics aberrations and response. The rarity and, in several cases, cryptic translocations require high qualification of the reference laboratories to provide reliable results within a short time frame.

Today, measurement of residual disease by immunophenotyping is the most appropriate method to define initial treatment response. The ongoing treatment protocols use residual disease detection either by immunophenotyping only or in combination with morphology for treatment stratification. The different response kinetics of fusion genes (KMT2A; AML1/Eto, CBL/M) and mutations (NPM1, FLT3-ITD, WT1), measured by quantitative PCR, makes this approach suitable and prognostically relevant only in some subgroups, such as PML/RARA. However, continuous monitoring after remission allows the early detection of molecular relapse. Although it is not entirely proven yet, the treatment of molecular relapse might be feasible with less intensive chemotherapy as bridge to transplant option. The international AmoRe 2017 trial (conducted by GPOH as sponsor) should allow alloHSCT without toxic re-induction in children with a molecular relapse by applying the epigenetically-effective low dose azacytidine. A reduction of MRD -levels to less than 10^−3^ should allow direct alloHSCT.

### 3.3. Myeloid Leukemia of Down Syndrome (ML-DS)

Until almost the end of the 20th century, patients with AML and Down syndrome (DS) were treated identically with the whole group of pediatric AML [63]. In the NOPHO AML-93, after the same treatment, a better 5-year survival was obsereved [64]. In CCG Studies 2861 and 2981, a significantly better 4-year-EFS and no benefit of the BMT was achieved in the patients with DS [65]. A significantly lower relapse rate was noticed in the MRC AML10 study and the BFM-83 and 98 studies [66,67]. Consequently, the therapeutic schema was modified to minimize the toxicities for this favorable group. In the AML02 Trial (JCCSG) patients with ML-DS were treated separately, and a stratification depended on the response after the inductions were implemented [68,69]. In the MRC AML 12, they were allocated for only four courses of chemotherapy and were not eligible for alloHSCT [70]. In the AML-BFM-93 study, the treatment of the DS patients included reduced doses of anthracycline and no high-dose cytarabine/mitoxantrone or cranial irradiation [71]. Since then, these patients have been treated with lower doses of chemotherapy. No maintenance therapy is recommended. Contrary to the excellent response and survival in the case newly diagnosed ML-DS, the overall survival after a relapse, which affects less than 10% of those patients, remains disappointing [65,72].

### 3.4. Supportive Care

All the improvements of the recent decades would be impossible without the progress in supportive care. The introduction of prophylactic antimycotic and, effective antibiotic regimens as well as improved intensive care, including sufficient, sensitive, and specific microbiologic diagnostics, enables intensive treatment with a limited rate of treatment-related death and toxicity [73,74,75]. In addition, recent data confirmed that strict separation of children under immunosuppressive therapy might not be required. The best strategy could be to react immediately with a very high level of awareness and structures in pediatric oncology sites [76]. Unfortunately, these structures are only given in some developed countries. 

### 3.5. Long-Term Toxicities

Pediatric AML and intensive treatment are associated with relevant long-term sequelae. All organ systems could be involved. Although much attention has been spent on cardiotoxicities, especially anthracycline-induced cardiomyopathy, severe damages of the liver, renal function, and endocrinology must be considered. Recent data showed the increased risk of early-onset cardiovascular diseases, neurology, and mental diseases [68]. In addition, treatment-induced malignancies occur in 2 to 5% within 10 to 20 years post-treatment. Unfortunately, to date, there is no plateau of the cumulative incidence [77].

## 4. Future

The therapy of pediatric AML is still based on intensive chemotherapy and, if necessary, alloHSCT. Despite significantly improved survival chances, this therapy has severe acute and long-term side effects [68,78,79]. Therapy-related toxicity is also relatively high at 2 to 4% [75] The aim of new, innovative therapies must be a more targeted treatment, presumably with fewer side effects, without jeopardizing achieved the results. Another aspect is that cure has a the highest priority. While, in adult disease with an age peak above 70 years, it may be beneficial to gain control of the disease for several years, cure must remain the primary aim in children. 

Early deaths from AML in children and adolescents continue to be a significant problem [80,81,82]. While, in some cases, the course is fateful due to the disease dynamics, on the other hand, a higher awareness of pediatricians, general practitioners, and pediatric hospitals could rescue some children. This is especially true for APL and monoblastic leukemia. These must be considered acute emergencies and treated immediately in cooperation with an experienced pediatric oncology center. Effective, quality-assuring structures (central consultation, reference laboratories), as established in some European countries, improve the chances of survival [83].

Several achievements will allow more reliable and precise diagnostics, mainly based on NGS, genome mapping, RNA seq, acetylation/methylation assays, and molecular single-cell characterization [84,85,86]. The challenge will be to integrate the complex data into meaningful results, allowing clinical decisions, better stratification, and more precise treatments. The reliable measurement of MRD by NGS-based approaches will cover all patients and give better insights into the fate of leukemic stem cells, clonal hierarchies, and evolution [86,87,88,89].

Regarding more precise treatment options, the differentiating therapies with all-trans retinoic acid (ATRA) and arsenic trioxide (ATO) in APL are already in use. For the first time, this allows the cure without chemotherapy and with significantly reduced side effects [90]. 

The use of antigen-mediated therapies was successful, especially with the CD33-specific and ozogamicin-coupled antibody GO (Mylotarg) [91]. However, even the positive randomized trials in adults and children have not yet led to a general marketing authorization in pediatric AML [5]. Other specific-acting agents, such as FLT3 or IDH1/2 inhibitors, have also shown efficacy in children and adolescents. Still, it has not been conclusively investigated whether this improves the chances of a cure or only means an effective but transient blast reduction [61]. The same is probably true for epigenetic approaches, which have been used very successfully in older adults. Combining BCL-2 inhibitors (venetoclax) with low-dose, mainly epigenetically active chemotherapy (e.g.azacytidine) modifies the clinical course of myelodysplastic syndromes/AML, especially with low proliferation activity, very positively with significantly improved survival [92]. Other approaches combined venetoclax with MDM2 or FLT3-ITD inhibitors; however, experience in children is lacking. The addition of venetoclax to high-dose cytarabine with or without idarubicin revealed a promising overall response of 69% [93]. Nevertheless, the contribution of venetoclax to this response rate needs to be evaluated. In summary, the relevance of venetoclax in pediatric AML needs to be confirmed in further trials (such as planned within the LLS-PedAL initiative). 

Another complex area of AML therapy includes various immunotherapies. One approach will be post-HSCT immunomodulation. The effectiveness has already been shown in post-HSCT treatment with donor-lymphocyte infusions (DLI) but definitively provides more options. Cytokine-induced killer (CIK) cells have already been introduced to clinical trials [94]. Other approaches use activated NK-cells [95]. In particular, the combination with immunomodulatory agents that optimize cellular treatments shows promising efficacy in preclinical and initial clinical studies. Directly related to these approaches are the current research findings on the importance of the microenvironment [96], its interaction with leukemic blasts, and the effects it induces on the selective proliferation of malignant cells, support of escape mechanism of leukemic stem cells, and inhibition of immunocompetence of effector cells, such as T/NK cells [97].

The successful cellular therapy approaches in B-cell lymphocytic leukemia/lymphomas raise high hopes for myeloid neoplasms [98]. However, the challenge in pediatric AML is more complex, while it is relatively easy to compensate for B lymphocyte eradication with antibody substitution, the reconstitution of myelopoiesis is only feasible with alloHSCT. Accordingly, to date, these therapeutic options must be viewed primarily as “bridge-to-transplant” regime. Nevertheless, cellular treatment options, such as gene-engineered T-CAR or NK-CAR cells, are promising approaches to enable more precise and hopefully less side-effective therapy in the future [99,100,101] Several targets have been addressed so far (CD33, CD123, CLL1, and others) [101,102,103]. 

Overall, it is unlikely that there will be “the one” effective therapy for a heterogeneous disease like AML. Only the optimized combination of all available options will allow a further, significant improvement of cure rates so that likely different treatments adapted to the AML subtype are needed. This underlines the need for further comprehensive research into the mechanisms of leukemogenesis, specific therapies, and, above all, systematic clinical research to develop scientifically-validated treatments, despite the small number of cases. This will only be possible if the international collaboration between the study groups will be further improved on a global level. It is important to learn more about small subgroups and more precise treatment, as well as reducing inequalities between countries and continents. The recently established Leukemia & Lymphoma Society (LLS), Pediatric Acute Leukemia (PedAL) initiative and the European Pediatric Acute Leukemia (EuPAL) foundation are on the way to launching such a platform in North America, Europe, Australia, and, hopefully, in Japan [3,104].

In this context, the therapies of AML in children, even if they have low economic impact, should not be considered exclusively as a “waste product” of adult medicine but should have a right to their own, child-specific therapy development. This applies to both, research on pediatric therapies and the timely establishment of therapies that have been successfully used in adult AML.

## Figures and Tables

**Table 1:** Risk Group definition by genetics and response, an example from the AIEOP-BFM AML 2020 Study.

Risk Group	Genetic Risk Criteria	Response Criteria
Standard Risk (SR)	CBFβ abnormalities t(8;21)(q22;q22) with adequate (≥2 log) reduction by qPCR at IND2 inv(16)(p13q22)/t(16;16)(p13;q22) Biallelic CEBPα aberrations t(16;21) CBFA2T3/RUNX1 *and* FLT3-ITD negative	Genetic standard risk and MRD < 0.1% at IND 2 t(8;21) and MRD > 2 log reduction at IND 2 (qPCR)
Intermediate Risk (IR)	NON SR and NON HR patients	Genetic standard or intermediate risk and MRD at IND 1 ≥ 0.1% and <1% and MRD at IND 2 < 0.1%
High Risk (HR)	Complex karyotype (≥3 aberrations including at least one structural aberration) *excluding those with recurrent translocations* Monosomal Karyotype, i.e., -7, -5/del(5q) 11q23/KMT2A rearrangements involving: t(4;11)(q21;q23) KMT2A/AFF1 t(6;11)(q27;q23) KMT2A/AFDN t(10;11)(p12;q23) KMT2A/MLLT10 t(9;11)(p21;q23) KMT2A/MLLT3 with other cytogenetic aberrations t(16;21)(p11;q22) FUS/ERG t(9;22)(q34;q11.2) BCR/ABL1 t(6;9)(p22;q34) DEK/NUP214 t(7;12)(q36;p13) MNX1/ETV6 inv3(q21q26)/t(3;3)(q21;q26) RPN1/MECOM 12p abnormalities$break$ FLT3-ITD with AR ≥ 0.5 not in combination with other recurrent abnormalities or NPM1 mutations WT1 mutation and FLT3-ITD inv(16)(p13q24) CBFA2T3/GLIS2 t(5;11)(q35;p15.5) NUP98/NSD1 and t(11;12)(p15;p13) NUP98/KDM5A Pure Erythroid leukemia	MRD ≥ 1% at IND 1 or ≥0.1% at IND 2 or (only if FLOW-result not available/informative) blast count ≥5% at IND 1

## Appendix A

**Table A1:** The outcome of pediatric AML. Clinical trials by the Cooperative Study Groups.

Study Group	Study	Periode	Patients (N)	EFS (%)	OS (%)	Relapse (%)	Source
AIEOP	AML2002/01	2002–2011	482	8-years 55.0 ± 2.6	8-years 67.7 ± 2.4	24	Pession et al. 2013 [105]
AML-BFM	AML-BFM 2012	2012–2018	324	5-years 65 ± 3	5-years 82 ± 3	22	Waack et al. 2020 [106]
	AML-BFM 2004	2004–2010	521	5-years 55 ± 2	5-years 74 ± 2	29	Creutzig et al. 2013 [107]
COG	AAML03P1 AAML0531 AAML1031	2003–2005 2006–2010 2011–2016	340 1022 1097	3-years 53 ± 6 3-years 53.1 vs. 46.9 3-years 45.9 ± 3	3-years 66 ± 5 3-years 69.4 vs. 65.4 3-years 65.4 ± 3	33 ± 6 32.8 vs. 41.3 47.2	Cooper et al. 2012 [108] Gamis et al. 2014 [109] Aplenc et al. 2020 [110]
JACLS	AML99	2000–2002	240	5-years 61.6 ± 6.5	5-years 75.6 ± 5.3	32.2	Tsukimoto et al. 2009 [31]
JPLSG	AML05	2006–2010	443	3-years 54.3 ± 2.4	3-years 73.2 ± 2.3	30.3	Tomizawa et al. 2013 [32]
MRC	MRC AML12	1995–2002	564	10-years 54	10-years 63	32	Gibson et al. 2011 [27]
	MRC AML 17	2010–2014			5-years 74		Burnett et al. [111]
NOPHO	NOPHO AML 2004	2004–2009	151	3-years 57 ± 5	3-years 69 ± 5	30	Abrahamsson et al. 2011 [7] Hasle et al. 2012 [29]
^99^PPLLSG	PPLLSG AML-98 AML-BFM 2012	1998–2002	195	5-years 46 ± 5	5-years 53 ± 5	24	Dluzniewska et al. 2010 [112] Czogala et al. 2021 [113]
						27	
		2015–2019	131	3-years 67 ± 5	3 years 75 ± 5	17	
SJCRH	AML02 AML08	2002–2008 2008–2017	216 285	3-years 61 3-years 52.9	3-years 71 3-years 74.8	21	Rubnitz et al. 2010 [30] Rubnitz et al. 2019 [114]

AIEOP (Associazione Italiana di Ematologia e Oncologia Pediatrica), AML-BFM (Berlin, Frankfurt, Münster), COG (Childhood Oncology Group), JACLS (Japanese Association of Childhood Leukemia Study), JPLSG (Japanese Pediatric Leukemia Study Group), NOPHO (Nordic Society for Pediatric Hematology and Oncology), MRC (Medical Research Council), PPLLSG (Polish Pediatric Leukemia/Lymphoma Study Group), SJCRH (St. Jude Children’s Research Hospital).

## References

[1] Bonaventure A., Harewood R., Stiller C.A., Gatta G., Clavel J., Stefan D.C., Carreira H., Spika D., Marcos-Gragera R., Peris-Bonet R. (2017). Worldwide comparison of survival from childhood leukaemia for 1995–2009, by subtype, age, and sex (CONCORD-2): A population-based study of individual data for 89,828 children from 198 registries in 53 countries. Lancet Haematol..

[2] Elgarten C.W., Aplenc R. (2020). Pediatric acute myeloid leukemia: Updates on biology, risk stratification, and therapy. Curr. Opin. Pediatr..

[3] Plana A., Furner B., Palese M., Dussault N., Birz S., Graglia L., Kush M., Nicholson J., Hecker-Nolting S., Gaspar N. (2021). Pediatric Cancer Data Commons: Federating and Democratizing Data for Childhood Cancer Research. JCO Clin. Cancer Inform..

[4] Zwaan C.M., Kolb E.A., Reinhardt D., Abrahamsson J., Adachi S., Aplenc R., de Bont E.S.J.M., de Moerloose B., Dworzak M., Gibson B.E.S. (2015). Collaborative Efforts Driving Progress in Pediatric Acute Myeloid Leukemia. J. Clin. Oncol..

[5] Creutzig U., van den Heuvel-Eibrink M.M., Gibson B., Dworzak M.N., Adachi S., de Bont E., Harbott J., Hasle H., Johnston D., Kinoshita A. (2012). Diagnosis and management of acute myeloid leukemia in children and adolescents: Recommendations from an international expert panel. Blood.

[6] Pollard J.A., Guest E., Alonzo T.A., Gerbing R.B., Loken M.R., Brodersen L.E., Kolb E.A., Aplenc R., Meshinchi S., Raimondi S.C. (2021). Gemtuzumab Ozogamicin Improves Event-Free Survival and Reduces Relapse in Pediatric KMT2A-Rearranged AML: Results From the Phase III Children’s Oncology Group Trial AAML0531. J. Clin. Oncol..

[7] Gurnari C., Voso M.T., Girardi K., Mastronuzzi A., Strocchio L. (2021). Acute Promyelocytic Leukemia in Children: A Model of Precision Medicine and Chemotherapy-Free Therapy. Int. J. Mol. Sci..

[8] Abrahamsson J., Forestier E., Heldrup J., Jahnukainen K., Jónsson O.G., Lausen B., Palle J., Zeller B., Hasle H. (2011). Response-guided induction therapy in pediatric acute myeloid leukemia with excellent remission rate. J. Clin. Oncol..

[9] Rasche M., Steidel E., Kondryn D., von Neuhoff N., Sramkova L., Creutzig U., Dworzak M., Reinhardt D. (2019). Impact of a Risk-Adapted Treatment Approach in Pediatric AML: A Report of the AML-BFM Registry 2012. Blood.

[10] Bennett J.M., Catovsky D., Daniel M.T., Flandrin G., Galton D.A., Gralnick H.R., Sultan C. (1976). Proposals for the classification of the acute leukaemias. French-American-British (FAB) co-operative group. Br. J. Haematol..

[11] Bennett J.M., Catovsky D., Daniel M.T., Flandrin G., Galton D.A., Gralnick H.R., Sultan C. (1985). Proposed revised criteria for the classification of acute myeloid leukemia. A report of the French-American-British Cooperative Group. Ann. Intern. Med..

[12] Bennett J.M., Catovsky D., Daniel M.T., Flandrin G., Galton D.A., Gralnick H.R., Sultan C. (1991). Proposal for the recognition of minimally differentiated acute myeloid leukaemia (AML-MO). Br. J. Haematol..

[13] Bloomfield C.D., Brunning R.D. (1985). FAB M7: Acute megakaryoblastic leukemia--beyond morphology. Ann. Intern. Med..

[14] Lee E.J., Pollak A., Leavitt R.D., Testa J.R., Schiffer C.A. (1987). Minimally differentiated acute nonlymphocytic leukemia: A distinct entity. Blood.

[15] Boiron M., Jacquillat C., Weil M., Tanzer J., Levy D., Sultan C., Bernard J. (1969). DAUNORUBICIN IN THE TREATMENT OF ACUTE MYELOCYTIC LEUKÆMIA. Lancet.

[16] Pizzo P.A., Henderson E.S., Leventhal B.G. (1976). Acute myelogenous leukemia in children: A preliminary report of combination chemotherapy. J. Pediatr..

[17] Wells R.J., Feusner J., Devney R., Woods W.G., Provisor A.J., Cairo M.S., Odom L.F., Nachman J., Jones G.R., Ettinger L.J. (1985). Sequential high-dose cytosine arabinoside-asparaginase treatment in advanced childhood leukemia. J. Clin. Oncol..

[18] Creutzig U., Ritter J., Langermann H.J., Riehm H., Henze G., Niethammer D., Jürgens H., Stollmann B., Lasson U., Kabisch H. (1983). Acute myelogenous leukemia in children: Results of the cooperative BFM-78 therapy study after 3 3/4 years. Klin. Padiatr..

[19] Steuber C.P., Humphrey G.B., McMillan C.W., Vietti T.J. (1978). Remission induction in acute myelogenous leukemia using cytosine arabinoside synchronization: A Southwest Oncology Group Study. Med. Pediatr. Oncol..

[20] Rees J. (1986). Principal results of the medical research council’s 8th acute myeloid leukaemia trial. Lancet.

[21] Stevens R.F., Hann I.M., Wheatley K., Gray R. (1992). Intensive chemotherapy with or without additional bone marrow transplantation in paediatric AML: Progress report on the MRC AML 10 trial. Medical Research Council Working Party on Childhood Leukaemia. Leukemia.

[22] Swirsky D.M., Li Y.S., Matthews J.G., Flemans R.J., Rees J.K., Hayhoe F.G. (1984). 8;21 translocation in acute granulocytic leukaemia: Cytological, cytochemical and clinical features. Br. J. Haematol..

[23] Creutzig U., Ritter J., Niederbiermann-Koczy G., Harbott J., Schellong G. (1989). Prognostische Bedeutung der Eosinophilie bei Kindern mit akuter myeloischer Leukämie in den Studien AML-BFM-78 und -83. Klin. Padiatr..

[24] Creutzig U., Ritter J., Schellong G. (1990). Identification of two risk groups in childhood acute myelogenous leukemia after therapy intensification in study AML-BFM-83 as compared with study AML-BFM-78. AML-BFM Study Group. Blood.

[25] Perel Y., Auvrignon A., Leblanc T., Michel G., Reguerre Y., Vannier J.-P., Dalle J.-H., Gandemer V., Schmitt C., Méchinaud F. (2005). Treatment of childhood acute myeloblastic leukemia: Dose intensification improves outcome and maintenance therapy is of no benefit--multicenter studies of the French LAME (Leucémie Aiguë Myéloblastique Enfant) Cooperative Group. Leukemia.

[26] Creutzig U., Zimmermann M., Reinhardt D., Rasche M., von Neuhoff C., Alpermann T., Dworzak M., Perglerová K., Zemanova Z., Tchinda J. (2016). Changes in cytogenetics and molecular genetics in acute myeloid leukemia from childhood to adult age groups. Cancer.

[27] Creutzig U., Ritter J., Zimmermann M., Hermann J., Gadner H., Sawatzki D.B., Niemeyer C.M., Schwabe D., Selle B., Boos J. (2001). Idarubicin improves blast cell clearance during induction therapy in children with AML: Results of study AML-BFM 93. AML-BFM Study Group. Leukemia.

[28] Gibson B.E.S., Webb D.K.H., Howman A.J., de Graaf S.S.N., Harrison C.J., Wheatley K. (2011). Results of a randomized trial in children with Acute Myeloid Leukaemia: Medical research council AML12 trial. Br. J. Haematol..

[29] Lie S.O., Abrahamsson J., Clausen N., Forestier E., Hasle H., Hovi L., Jonmundsson G., Mellander L., Siimes M.A., Yssing M. (2005). Long-term results in children with AML: NOPHO-AML Study Group--report of three consecutive trials. Leukemia.

[30] Hasle H., Abrahamsson J., Forestier E., Ha S.-Y., Heldrup J., Jahnukainen K., Jónsson Ó.G., Lausen B., Palle J., Zeller B. (2012). Gemtuzumab ozogamicin as postconsolidation therapy does not prevent relapse in children with AML: Results from NOPHO-AML 2004. Blood.

[31] Rubnitz J.E., Inaba H., Dahl G., Ribeiro R.C., Bowman W.P., Taub J., Pounds S., Razzouk B.I., Lacayo N.J., Cao X. (2010). Minimal residual disease-directed therapy for childhood acute myeloid leukaemia: Results of the AML02 multicentre trial. Lancet Oncol..

[32] Tsukimoto I., Tawa A., Horibe K., Tabuchi K., Kigasawa H., Tsuchida M., Yabe H., Nakayama H., Kudo K., Kobayashi R. (2009). Risk-stratified therapy and the intensive use of cytarabine improves the outcome in childhood acute myeloid leukemia: The AML99 trial from the Japanese Childhood AML Cooperative Study Group. J. Clin. Oncol..

[33] Tomizawa D., Tawa A., Watanabe T., Saito A.M., Kudo K., Taga T., Iwamoto S., Shimada A., Terui K., Moritake H. (2013). Appropriate dose reduction in induction therapy is essential for the treatment of infants with acute myeloid leukemia: A report from the Japanese Pediatric Leukemia/Lymphoma Study Group. Int. J. Hematol..

[34] Creutzig U., Reinhardt D. (2002). Current controversies: Which patients with acute myeloid leukaemia should receive a bone marrow transplantation?—A European view. Br. J. Haematol..

[35] Creutzig U., Diekamp S., Zimmermann M., Reinhardt D. (2007). Longitudinal evaluation of early and late anthracycline cardiotoxicity in children with AML. Pediatr. Blood Cancer.

[36] Gibson B.E.S., Wheatley K., Hann I.M., Stevens R.F., Webb D., Hills R.K., de Graaf S.S.N., Harrison C.J. (2005). Treatment strategy and long-term results in paediatric patients treated in consecutive UK AML trials. Leukemia.

[37] Klingebiel T., Ritter J., Schellong G., Creutzig U., Riehm H., Henze G., Bender-Götze C., Dopfer R., Ebell W., Friedrich W. (1991). Role and perspectives of BMT in AML: The BFM experience. Bone Marrow Transplant..

[38] Klusmann J.-H., Reinhardt D., Zimmermann M., Kremens B., Vormoor J., Dworzak M., Creutzig U., Klingebiel T. (2012). The role of matched sibling donor allogeneic stem cell transplantation in pediatric high-risk acute myeloid leukemia: Results from the AML-BFM 98 study. Haematologica.

[39] Locatelli F., Masetti R., Rondelli R., Zecca M., Fagioli F., Rovelli A., Messina C., Lanino E., Bertaina A., Favre C. (2015). Outcome of children with high-risk acute myeloid leukemia given autologous or allogeneic hematopoietic cell transplantation in the aieop AML-2002/01 study. Bone Marrow Transplant..

[40] Lonetti A., Pession A., Masetti R. (2019). Targeted Therapies for Pediatric AML: Gaps and Perspective. Front. Pediatr..

[41] Woods W.G., Neudorf S., Gold S., Sanders J., Buckley J.D., Barnard D.R., Dusenbery K., DeSwarte J., Arthur D.C., Lange B.J. (2001). A comparison of allogeneic bone marrow transplantation, autologous bone marrow transplantation, and aggressive chemotherapy in children with acute myeloid leukemia in remission. Blood.

[42] Goldman F.D., Rumelhart S.L., DeAlacron P., Holida M.D., Lee N.F., Miller J., Trigg M., Giller R. (2000). Poor outcome in children with refractory/relapsed leukemia undergoing bone marrow transplantation with mismatched family member donors. Bone Marrow Transplant..

[43] Sung L., Buckstein R., Doyle J.J., Crump M., Detsky A.S. (2003). Treatment options for patients with acute myeloid leukemia with a matched sibling donor: A decision analysis. Cancer.

[44] Chen A.R., Alonzo T.A., Woods W.G., Arceci R.J. (2002). Current controversies: Which patients with acute myeloid leukaemia should receive a bone marrow transplantation?—An American view. Br. J. Haematol..

[45] Wheatley K. (2002). Current controversies: Which patients with acute myeloid leukaemia should receive a bone marrow transplantation? A statistician’s view. Br. J. Haematol..

[46] Creutzig U., Ritter J., Zimmermann M., Schellong G. (1993). Does cranial irradiation reduce the risk for bone marrow relapse in acute myelogenous leukemia? Unexpected results of the Childhood Acute Myelogenous Leukemia Study BFM-87. J. Clin. Oncol..

[47] Woods W.G., Kobrinsky N., Buckley J.D., Lee J.W., Sanders J., Neudorf S., Gold S., Barnard D.R., DeSwarte J., Dusenbery K. (1996). Timed-sequential induction therapy improves postremission outcome in acute myeloid leukemia: A report from the Children’s Cancer Group. Blood.

[48] Pession A., Rondelli R., Basso G., Rizzari C., Testi A.M., Fagioli F., de Stefano P., Locatelli F. (2005). Treatment and long-term results in children with acute myeloid leukaemia treated according to the AIEOP AML protocols. Leukemia.

[49] Creutzig U., Büchner T., Sauerland M.C., Zimmermann M., Reinhardt D., Döhner H., Schlenk R.F. (2008). Significance of age in acute myeloid leukemia patients younger than 30 years: A common analysis of the pediatric trials AML-BFM 93/98 and the adult trials AMLCG 92/99 and AMLSG HD93/98A. Cancer.

[50] Wells R.J., Woods W.G., Buckley J.D., Odom L.F., Benjamin D., Bernstein I., Betcher D., Feig S., Kim T., Ruymann F. (1994). Treatment of newly diagnosed children and adolescents with acute myeloid leukemia: A Childrens Cancer Group study. J. Clin. Oncol..

[51] Rasche M., Zimmermann M., Borschel L., Bourquin J.-P., Dworzak M., Klingebiel T., Lehrnbecher T., Creutzig U., Klusmann J.-H., Reinhardt D. (2018). Successes and challenges in the treatment of pediatric acute myeloid leukemia: A retrospective analysis of the AML-BFM trials from 1987 to 2012. Leukemia.

[52] Langebrake C., Creutzig U., Dworzak M., Hrusak O., Mejstrikova E., Griesinger F., Zimmermann M., Reinhardt D. (2006). Residual disease monitoring in childhood acute myeloid leukemia by multiparameter flow cytometry: The MRD-AML-BFM Study Group. J. Clin. Oncol..

[53] Rubnitz J.E., Crews K.R., Pounds S., Yang S., Campana D., Gandhi V.V., Raimondi S.C., Downing J.R., Razzouk B.I., Pui C.-H. (2009). Combination of cladribine and cytarabine is effective for childhood acute myeloid leukemia: Results of the St Jude AML97 trial. Leukemia.

[54] Tierens A., Bjørklund E., Siitonen S., Marquart H.V., Wulff-Juergensen G., Pelliniemi T.-T., Forestier E., Hasle H., Jahnukainen K., Lausen B. (2016). Residual disease detected by flow cytometry is an independent predictor of survival in childhood acute myeloid leukaemia; results of the NOPHO-AML 2004 study. Br. J. Haematol..

[55] van der Velden V.H.J., van der Sluijs-Geling A., Gibson B.E.S., te Marvelde J.G., Hoogeveen P.G., Hop W.C.J., Wheatley K., Bierings M.B., Schuurhuis G.J., de Graaf S.S.N. (2010). Clinical significance of flowcytometric minimal residual disease detection in pediatric acute myeloid leukemia patients treated according to the DCOG ANLL97/MRC AML12 protocol. Leukemia.

[56] Inaba H., Coustan-Smith E., Cao X., Pounds S.B., Shurtleff S.A., Wang K.Y., Raimondi S.C., Onciu M., Jacobsen J., Ribeiro R.C. (2012). Comparative analysis of different approaches to measure treatment response in acute myeloid leukemia. J. Clin. Oncol..

[57] Rasche M., Zimmermann M., Steidel E., Alonzo T., Aplenc R., Bourquin J.-P., Boztug H., Cooper T., Gamis A.S., Gerbing R.B. (2021). Survival Following Relapse in Children with Acute Myeloid Leukemia: A Report from AML-BFM and COG. Cancers.

[58] Rasche M., Steidel E., Zimmermann M., Bourquin J.-P., Boztug H., Janotova I., Kolb E.A., Lehrnbecher T., von Neuhoff N., Niktoreh N. (2021). Second Relapse of Pediatric Patients with Acute Myeloid Leukemia: A Report on Current Treatment Strategies and Outcome of the AML-BFM Study Group. Cancers.

[59] Reinhardt D., von Neuhoff C., Sander A., Creutzig U. (2012). Prognostische Relevanz genetischer Aberrationen der akuten myeloischen Leukämie bei Kindern und Jugendlichen. Klin. Padiatr..

[60] Sauer M.G., Lang P.J., Albert M.H., Bader P., Creutzig U., Eyrich M., Greil J., Gruhn B., Holter W., Klingebiel T. (2020). Hematopoietic stem cell transplantation for children with acute myeloid leukemia-results of the AML SCT-BFM 2007 trial. Leukemia.

[61] Kalwak K., Mielcarek M., Patrick K., Styczynski J., Bader P., Corbacioglu S., Burkhardt B., Sykora K.W., Drabko K., Gozdzik J. (2020). Treosulfan-fludarabine-thiotepa-based conditioning treatment before allogeneic hematopoietic stem cell transplantation for pediatric patients with hematological malignancies. Bone Marrow Transplant..

[62] Inaba H., Rubnitz J.E., Coustan-Smith E., Li L., Furmanski B.D., Mascara G.P., Heym K.M., Christensen R., Onciu M., Shurtleff S.A. (2011). Phase I pharmacokinetic and pharmacodynamic study of the multikinase inhibitor sorafenib in combination with clofarabine and cytarabine in pediatric relapsed/refractory leukemia. J. Clin. Oncol..

[63] de Rooij J.D.E., Branstetter C., Ma J., Li Y., Walsh M.P., Cheng J., Obulkasim A., Dang J., Easton J., Verboon L.J. (2017). Pediatric non-Down syndrome acute megakaryoblastic leukemia is characterized by distinct genomic subsets with varying outcomes. Nat. Genet..

[64] Abildgaard L., Ellebaek E., Gustafsson G., Abrahamsson J., Hovi L., Jonmundsson G., Zeller B., Hasle H. (2006). Optimal treatment intensity in children with Down syndrome and myeloid leukaemia: Data from 56 children treated on NOPHO-AML protocols and a review of the literature. Ann. Hematol..

[65] Hitzler J.K., He W., Doyle J., Cairo M., Camitta B.M., Chan K.W., Diaz Perez M.A., Fraser C., Gross T.G., Horan J.T. (2013). Outcome of transplantation for acute myelogenous leukemia in children with Down syndrome. Biol. Blood Marrow Transplant..

[66] Flasinski M., Scheibke K., Zimmermann M., Creutzig U., Reinhardt K., Verwer F., de Haas V., van der Velden V.H.J., von Neuhoff C., Zwaan C.M. (2018). Low-dose cytarabine to prevent myeloid leukemia in children with Down syndrome: TMD Prevention 2007 study. Blood Adv..

[67] Rao A., Hills R.K., Stiller C., Gibson B.E., de Graaf S.S.N., Hann I.M., O’Marcaigh A., Wheatley K., Webb D.K.H. (2006). Treatment for myeloid leukaemia of Down syndrome: Population-based experience in the UK and results from the Medical Research Council AML 10 and AML 12 trials. Br. J. Haematol..

[68] Bhatt N.S., Baassiri M.J., Liu W., Bhakta N., Chemaitilly W., Ehrhardt M.J., Inaba H., Krull K., Ness K.K., Rubnitz J.E. (2021). Late outcomes in survivors of childhood acute myeloid leukemia: A report from the St. Jude Lifetime Cohort Study. Leukemia.

[69] Mast K.J., Taub J.W., Alonzo T.A., Gamis A.S., Mosse C.A., Mathew P., Berman J.N., Wang Y.-C., Jones H.M., Campana D. (2020). Pathologic Features of Down Syndrome Myelodysplastic Syndrome and Acute Myeloid Leukemia: A Report From the Children’s Oncology Group Protocol AAML0431. Arch. Pathol. Lab. Med..

[70] Sussman R.T., Manning B., Ackerman D., Bigdeli A., Pammer P., Velu P.D., Luger S.M., Bagg A., Carroll M., Morrissette J.J.D. (2021). Interpretative differences of combined cytogenetic and molecular profiling highlights differences between MRC and ELN classifications of AML. Cancer Genet..

[71] Creutzig U., Reinhardt D., Diekamp S., Dworzak M., Stary J., Zimmermann M. (2005). AML patients with Down syndrome have a high cure rate with AML-BFM therapy with reduced dose intensity. Leukemia.

[72] Uffmann M., Rasche M., Zimmermann M., von Neuhoff C., Creutzig U., Dworzak M., Scheffers L., Hasle H., Zwaan C.M., Reinhardt D. (2017). Therapy reduction in patients with Down syndrome and myeloid leukemia: The international ML-DS 2006 trial. Blood.

[73] Bochennek K., Hassler A., Perner C., Gilfert J., Schöning S., Klingebiel T., Reinhardt D., Creutzig U., Lehrnbecher T. (2016). Infectious complications in children with acute myeloid leukemia: Decreased mortality in multicenter trial AML-BFM 2004. Blood Cancer J..

[74] Lehrnbecher T., Kaiser J., Varwig D., Ritter J., Groll A.H., Creutzig U., Klingebiel T., Schwabe D. (2007). Antifungal usage in children undergoing intensive treatment for acute myeloid leukemia: Analysis of the multicenter clinical trial AML-BFM 93. Eur. J. Clin. Microbiol. Infect. Dis..

[75] Lehrnbecher T., Varwig D., Kaiser J., Reinhardt D., Klingebiel T., Creutzig U. (2004). Infectious complications in pediatric acute myeloid leukemia: Analysis of the prospective multi-institutional clinical trial AML-BFM 93. Leukemia.

[76] Tramsen L., Salzmann-Manrique E., Bochennek K., Klingebiel T., Reinhardt D., Creutzig U., Sung L., Lehrnbecher T. (2016). Lack of Effectiveness of Neutropenic Diet and Social Restrictions as Anti-Infective Measures in Children With Acute Myeloid Leukemia: An Analysis of the AML-BFM 2004 Trial. J. Clin. Oncol..

[77] Bhakta N., Liu Q., Ness K.K., Baassiri M., Eissa H., Yeo F., Chemaitilly W., Ehrhardt M.J., Bass J., Bishop M.W. (2017). The cumulative burden of surviving childhood cancer: An initial report from the St Jude Lifetime Cohort Study (SJLIFE). Lancet.

[78] Molgaard-Hansen L., Glosli H., Jahnukainen K., Jarfelt M., Jónmundsson G.K., Malmros-Svennilson J., Nysom K., Hasle H. (2011). Quality of health in survivors of childhood acute myeloid leukemia treated with chemotherapy only: A NOPHO-AML study. Pediatr. Blood Cancer.

[79] Stefanski K.J., Anixt J.S., Goodman P., Bowers K., Leisenring W., Scott Baker K., Burns K., Howell R., Davies S., Robison L.L. (2021). Long-Term Neurocognitive and Psychosocial Outcomes After Acute Myeloid Leukemia: A Childhood Cancer Survivor Study Report. JNCI J. Natl. Cancer Inst..

[80] Lins M.M., Mello M.J.G., Ribeiro R.C., de Camargo B., de Fátima Pessoa Militão Albuquerque M., Thuler L.C.S. (2019). Survival and risk factors for mortality in pediatric patients with acute myeloid leukemia in a single reference center in low-middle-income country. Ann. Hematol..

[81] Creutzig U., Zimmermann M., Reinhardt D., Dworzak M., Stary J., Lehrnbecher T. (2004). Early deaths and treatment-related mortality in children undergoing therapy for acute myeloid leukemia: Analysis of the multicenter clinical trials AML-BFM 93 and AML-BFM 98. J. Clin. Oncol..

[82] Abla O., Angelini P., Di Giuseppe G., Kanani M.F., Lau W., Hitzler J., Sung L., Naqvi A. (2016). Early Complications of Hyperleukocytosis and Leukapheresis in Childhood Acute Leukemias. J. Pediatr. Hematol. Oncol..

[83] Klein K., van Litsenburg R.R.L., de Haas V., Dors N., van den Heuvel-Eibrink M.M., Knops R.R.G., Tissing W.J.E., Versluys B.A., Zwaan C.M., Kaspers G.J.L. (2020). Causes of early death and treatment-related death in newly diagnosed pediatric acute myeloid leukemia: Recent experiences of the Dutch Childhood Oncology Group. Pediatr. Blood Cancer.

[84] Velten L., Story B.A., Hernández-Malmierca P., Raffel S., Leonce D.R., Milbank J., Paulsen M., Demir A., Szu-Tu C., Frömel R. (2021). Identification of leukemic and pre-leukemic stem cells by clonal tracking from single-cell transcriptomics. Nat. Commun..

[85] Walter C., Pozzorini C., Reinhardt K., Geffers R., Xu Z., Reinhardt D., von Neuhoff N., Hanenberg H. (2018). Single-cell whole exome and targeted sequencing in NPM1/FLT3 positive pediatric acute myeloid leukemia. Pediatr. Blood Cancer.

[86] Thol F., Gabdoulline R., Liebich A., Klement P., Schiller J., Kandziora C., Hambach L., Stadler M., Koenecke C., Flintrop M. (2018). Measurable residual disease monitoring by NGS before allogeneic hematopoietic cell transplantation in AML. Blood.

[87] Bertuccio S.N., Anselmi L., Masetti R., Lonetti A., Cerasi S., Polidori S., Serravalle S., Pession A. (2021). Exploiting Clonal Evolution to Improve the Diagnosis and Treatment Efficacy Prediction in Pediatric AML. Cancers.

[88] Chen X., Zhu H., Qiao C., Zhao S., Liu L., Wang Y., Jin H., Qian S., Wu Y. (2021). Next-generation sequencing reveals gene mutations landscape and clonal evolution in patients with acute myeloid leukemia. Hematology.

[89] Onecha E., Rapado I., Luz Morales M., Carreño-Tarragona G., Martinez-Sanchez P., Gutierrez X., Sáchez Pina J.M., Linares M., Gallardo M., Martinez-López J. (2021). Monitoring of clonal evolution of acute myeloid leukemia identifies the leukemia subtype, clinical outcome and potential new drug targets for post-remission strategies or relapse. Haematologica.

[90] Creutzig U., Dworzak M., von Neuhoff N., Rasche M., Reinhardt D. (2018). Akute Promyelozyten-Leukämie: Neue Behandlungsstrategien mit ATRA und ATO-AML-BFM-Empfehlungen. Klin. Padiatr..

[91] Zwaan C.M., Meshinchi S., Radich J.P., Veerman A.J.P., Huismans D.R., Munske L., Podleschny M., Hählen K., Pieters R., Zimmermann M. (2003). FLT3 internal tandem duplication in 234 children with acute myeloid leukemia: Prognostic significance and relation to cellular drug resistance. Blood.

[92] Winters A.C., Maloney K.W., Treece A.L., Gore L., Franklin A.K. (2020). Single-center pediatric experience with venetoclax and azacitidine as treatment for myelodysplastic syndrome and acute myeloid leukemia. Pediatr. Blood Cancer.

[93] Karol S.E., Alexander T.B., Budhraja A., Pounds S.B., Canavera K., Wang L., Wolf J., Klco J.M., Mead P.E., Das Gupta S. (2020). Venetoclax in combination with cytarabine with or without idarubicin in children with relapsed or refractory acute myeloid leukaemia: A phase 1, dose-escalation study. Lancet Oncol..

[94] Merker M., Salzmann-Manrique E., Katzki V., Huenecke S., Bremm M., Bakhtiar S., Willasch A., Jarisch A., Soerensen J., Schulz A. (2019). Clearance of Hematologic Malignancies by Allogeneic Cytokine-Induced Killer Cell or Donor Lymphocyte Infusions. Biol. Blood Marrow Transplant..

[95] Nguyen R., Wu H., Pounds S., Inaba H., Ribeiro R.C., Cullins D., Rooney B., Bell T., Lacayo N.J., Heym K. (2019). A phase II clinical trial of adoptive transfer of haploidentical natural killer cells for consolidation therapy of pediatric acute myeloid leukemia. J. Immunother. Cancer.

[96] Sendker S., Waack K., Reinhardt D. (2021). Far from Health: The Bone Marrow Microenvironment in AML, A Leukemia Supportive Shelter. Children.

[97] Sendker S., Reinhardt D., Niktoreh N. (2021). Redirecting the Immune Microenvironment in Acute Myeloid Leukemia. Cancers.

[98] Maude S.L., Laetsch T.W., Buechner J., Rives S., Boyer M., Bittencourt H., Bader P., Verneris M.R., Stefanski H.E., Myers G.D. (2018). Tisagenlecleucel in Children and Young Adults with B-Cell Lymphoblastic Leukemia. N. Engl. J. Med..

[99] Morgan M.A., Kloos A., Lenz D., Kattre N., Nowak J., Bentele M., Keisker M., Dahlke J., Zimmermann K., Sauer M. (2021). Improved Activity against Acute Myeloid Leukemia with Chimeric Antigen Receptor (CAR)-NK-92 Cells Designed to Target CD123. Viruses.

[100] Tang X., Yang L., Li Z., Nalin A.P., Dai H., Xu T., Yin J., You F., Zhu M., Shen W. (2018). First-in-man clinical trial of CAR NK-92 cells: Safety test of CD33-CAR NK-92 cells in patients with relapsed and refractory acute myeloid leukemia. Am. J. Cancer Res..

[101] Zhang H., Wang P., Li Z., He Y., Gan W., Jiang H. (2021). Anti-CLL1 Chimeric Antigen Receptor T-Cell Therapy in Children with Relapsed/Refractory Acute Myeloid Leukemia. Clin. Cancer Res..

[102] Epperly R., Gottschalk S., Velasquez M.P. (2020). Harnessing T Cells to Target Pediatric Acute Myeloid Leukemia: CARs, BiTEs, and Beyond. Children.

[103] Pizzitola I., Anjos-Afonso F., Rouault-Pierre K., Lassailly F., Tettamanti S., Spinelli O., Biondi A., Biagi E., Bonnet D. (2014). Chimeric antigen receptors against CD33/CD123 antigens efficiently target primary acute myeloid leukemia cells in vivo. Leukemia.

[104] Pearson A.D.J., Zwaan C.M., Kolb E.A., Karres D., Guillot J., Kim S.Y., Marshall L., Tasian S.K., Smith M., Cooper T. (2020). Paediatric Strategy Forum for medicinal product development for acute myeloid leukaemia in children and adolescents: ACCELERATE in collaboration with the European Medicines Agency with participation of the Food and Drug Administration. Eur. J. Cancer.

[105] Pession A., Masetti R., Rizzari C., Putti M.C., Casale F., Fagioli F., Luciani M., Lo Nigro L., Menna G., Micalizzi C. (2013). Results of the AIEOP AML 2002/01 multicenter prospective trial for the treatment of children with acute myeloid leukemia. Blood.

[106] Waack K. (2020). Improved Outcome in Pediatric AML—THe AML-BFM 2012 Study. Blood.

[107] Creutzig U., Zimmermann M., Bourquin J.-P., Dworzak M.N., Fleischhack G., Graf N., Klingebiel T., Kremens B., Lehrnbecher T., von Neuhoff C. (2013). Randomized trial comparing liposomal daunorubicin with idarubicin as induction for pediatric acute myeloid leukemia: Results from Study AML-BFM 2004. Blood.

[108] Cooper T.M., Franklin J., Gerbing R.B., Alonzo T.A., Hurwitz C., Raimondi S.C., Hirsch B., Smith F.O., Mathew P., Arceci R.J. (2012). AAML03P1, a pilot study of the safety of gemtuzumab ozogamicin in combination with chemotherapy for newly diagnosed childhood acute myeloid leukemia: A report from the Children’s Oncology Group. Cancer.

[109] Gamis A.S., Alonzo T.A., Meshinchi S., Sung L., Gerbing R.B., Raimondi S.C., Hirsch B.A., Kahwash S.B., Heerema-McKenney A., Winter L. (2014). Gemtuzumab ozogamicin in children and adolescents with de novo acute myeloid leukemia improves event-free survival by reducing relapse risk: Results from the randomized phase III Children’s Oncology Group trial AAML0531. J. Clin. Oncol..

[110] Aplenc R., Meshinchi S., Sung L., Alonzo T., Choi J., Fisher B., Gerbing R., Hirsch B., Horton T., Kahwash S. (2020). Bortezomib with standard chemotherapy for children with acute myeloid leukemia does not improve treatment outcomes: A report from the Children’s Oncology Group. Haematologica.

[111] Burnett A.K., Hills R.K., Russell N. (2020). Twenty five years of UK trials in acute myeloid leukaemia: What have we learned?. Br. J. Haematol..

[112] Dłuzniewska A., Balwierz W., Balcerska A., Chybicka A., Kamieńska E., Karolczyk G., Karpińska-Derda I., Krawczuk-Rybak M., Kowalczyk J.R., Lewandowska D. (2010). Niepowodzenia leczenia w ostrej białaczce szpikowej u dzieci: Ponad 25-letnie doświadczenia Polskiej Pediatrycznej Grupy ds. Leczenia Białaczek i Chłoniaków. Przegl. Lek..

[113] Czogała M., Balwierz W., Pawińska-Wąsikowska K., Książek T., Bukowska-Strakova K., Czogała W., Sikorska-Fic B., Matysiak M., Skalska-Sadowska J., Wachowiak J. (2021). Advances in the First Line Treatment of Pediatric Acute Myeloid Leukemia in the Polish Pediatric Leukemia and Lymphoma Study Group from 1983 to 2019. Cancers.

[114] Rubnitz J.E., Lacayo N.J., Inaba H., Heym K., Ribeiro R.C., Taub J., McNeer J., Degar B., Schiff D., Yeoh A.E.-J. (2019). Clofarabine Can Replace Anthracyclines and Etoposide in Remission Induction Therapy for Childhood Acute Myeloid Leukemia: The AML08 Multicenter, Randomized Phase III Trial. J. Clin. Oncol..

